# Neuroimaging Correlates of Post‐Stroke Pain After Ischemic Stroke: Secondary Analysis of the INSPiRE‐TMS Trial

**DOI:** 10.1002/hbm.70602

**Published:** 2026-07-08

**Authors:** Alina Stegemann, Ana Sofia Rios, Ahmed Khalil, Ulrike Grittner, Uchralt Temuulen, Ramanan Ganeshan, Tim Bastian Braemswig, Andreas Horn, Thomas Ihl, Heinrich J. Audebert, Anna Kufner, Matthias Endres

**Affiliations:** ^1^ Charité – Universitätsmedizin Berlin Corporate Member of Freie Universität Berlin and Humboldt‐Universität Zu Berlin, Klinik für Neurologie Mit Experimenteller Neurologie Berlin Germany; ^2^ Charité – Universitätsmedizin Berlin, Corporate Member of Freie Universität Berlin and Humboldt‐Universität Zu Berlin Center for Stroke Research Berlin (CSB) Berlin Germany; ^3^ Berlin Institute of Health (BIH) at Charité Universitätsmedizin Berlin Berlin Germany; ^4^ Charité – Universitätsmedizin Berlin, Corporate Member of Freie Universität Berlin and Humboldt‐Universität Zu Berlin Institute of Biometry and Clinical Epidemiology Berlin Germany; ^5^ German Centre for Cardiovascular Research (Deutsches Zentrum für Herz Kreislauferkrankungen, DZHK) Berlin Germany; ^6^ Center for Brain Circuit Therapeutics Department of Neurology, Brigham & Women's Hospital Harvard Medical School Boston Massachusetts USA; ^7^ MGH Neurosurgery & Center for Neurotechnology and Neurorecovery (CNTR) at MGH Neurology Massachusetts General Hospital Harvard Medical School Boston Massachusetts USA; ^8^ German Center for Mental Health (Deutsches Zentrum für Psychische Gesundheit, DZPG) Berlin Germany; ^9^ German Center for Neurodegenerative Diseases (Deutsches Zentrum für Neurode‐ Generative Erkrankungen, DZNE) Berlin Germany

**Keywords:** chronic pain, lesion mapping, post‐stroke pain, stroke

## Abstract

Post‐stroke pain (PSP) affects nearly half of stroke survivors, severely compromising quality of life. The causes of PSP remain underexplored, although there is likely a complex interplay of lesion effects, psychological factors, and mobility that play a role in its development. The aim of the study was to investigate clinical characteristics associated with PSP, as well as structural and functional correlates of PSP using lesion symptom (LSM) and network mapping (LNM). We analyzed data from the INSPiRE‐TMS cohort, encompassing 1022 minor ischemic stroke patients. Pain severity and psychological factors (EQUATION 5D‐3L questionnaire) were assessed annually for up to 3 years. In a sub‐group of 391 patients with available imaging data, LSM and LNM analyses were conducted to identify neural correlates of PSP. Overall, 47% of patients reported pain 1‐year post‐stroke. Multivariable regression analyses identified baseline anxiety as associated with PSP assessed at 1‐year post‐stroke (OR 2.90, 95% CI 1.17–7.17, *p* = 0.021). LSM did not identify any voxels associated with new severe pain. LNM identified a network involving the anterior cingulate cortex, thalamus, and insular cortex. Adjusting for anxiety highlighted distinct network contributions, suggesting interactive effects of psychological states on pain perception. Automated comparison to large metanalytical findings using Neurosynth associated the terms ‘pain’ and ‘nociception’ most strongly to the identified network. PSP is closely associated with psychological factors such as anxiety. LNM of PSP revealed disruptions in a pain‐related neural network consistent with prior pain research. These results warrant external validation and could guide future network‐targeted neuromodulation therapies.

**Trial Registration:** ClinicalTrials.gov identifier: NCT01586702

## Key Points

- This secondary analysis of a prospective stroke cohort identified anxiety as a key factor associated with severe post‐stroke pain, whereas conventional stroke severity measures and lesion volume showed no independent association in the multivariable model.
- Lesion network mapping links new severe post‐stroke pain to disruptions in a distributed pain‐related network involving the anterior cingulate cortex, insula, and thalamus; adjustment for baseline anxiety yielded a more clearly delineated network pattern.
- Clinically relevant pain can occur even after mild ischemic stroke and may warrant proactive evaluation and treatment planning.
- Early screening for anxiety after ischemic stroke may help identify patients at risk of severe post‐stroke pain and support closer multidisciplinary follow‐up and pain management.
- Network‐level findings suggest that new severe pain following ischemic stroke may involve distributed pain‐processing circuits, including the anterior cingulate cortex, insula, and thalamus, which may inform future studies of neuromodulatory treatment strategies.

## Graphical abstract

In 1022 minor ischemic stroke patients, baseline anxiety was associated with new severe pain at 1 year. In the MRI subgroup (*N* = 391), lesion network mapping implicated a distributed network including the insula, anterior cingulate cortex, and thalamus; Neurosynth decoding showed the highest similarity to pain and nociception terms.

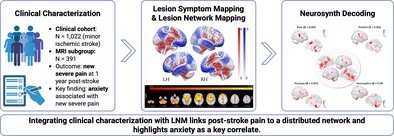

**Abbreviations**
ACC: anterior cingulate cortex
BOLD: blood‐oxygenation‐level dependent
DWI: diffusion‐weighted imaging
EQUATION 5D‐3L: European Quality of Life 5 Dimensions 3 Level Version
FLAIR: fluid‐attenuated inversion recovery
FWE: family wise error
INSPiRE‐TMS: intensified secondary prevention intending a reduction of recurrent events in TIA and minor stroke patients
LNM: lesion‐network mapping
LSM: lesion‐symptom mapping
MRI: magnetic resonance imaging
mRS: modified Rankin Scale
NIHSS: National Institutes of Health Stroke Scale
PSP: post‐stroke pain
rs‐fMRI: resting‐state functional magnetic resonance imaging
TFCE: threshold‐free cluster enhancement
TIA: transient ischemic attack
TOAST: Trial of Org 10172 in Acute Stroke Treatment
VPL: ventral posterolateral
VPM: ventral posteromedial

## 1 Introduction

Stroke is a major global health concern, profoundly impacting patients' quality of life. Among its various complications, post‐stroke pain (PSP) is particularly debilitating, affecting both physical and emotional well‐being. PSP has been linked to reduced mobility (Westerlind et al. 2020), independence (Ali et al. 2023), and difficulties with daily activities (Hansen et al. 2012), as well as increased anxiety, depression (Ali et al. 2023; Lundstrom et al. 2009; Payton and Soundy 2020), fatigue (Hoang et al. 2012) and overall decreased quality of life (Westerlind et al. 2020; Payton and Soundy 2020; Naess et al. 2012a). Recognizing the multifaceted nature of PSP is critical not only for accurate diagnosis but also for developing effective therapeutic strategies.

Clinical studies have found that the frequency of PSP is high, affecting up to 50% of stroke patients, although prevalence estimates vary depending on follow‐up timepoint, stroke severity, and pain definition (Hansen et al. 2012; Naess et al. 2012a). The onset of PSP can vary widely, occurring from 1 month up to 2 years post‐stroke, and persisting for up to 5 years (Westerlind et al. 2020). While PSP encompasses diverse etiologies—including spasticity, shoulder pain, central (neuropathic) pain, headache, and musculoskeletal pain (Hansen et al. 2012; O'Donnell et al. 2013)—its complexity extends beyond physical causes. Female sex, older age, depression, spasticity, reduced upper extremity mobility, and somatosensory impairment, among others, have been described as risk factors (O'Donnell et al. 2013; Harrison and Field 2015). However, reported associations differ across studies, likely reflecting heterogeneity in pain definition and stroke populations. Moreover, psychological factors such as anxiety and depression may influence the experience and intensity of PSP (Ali et al. 2023; Lundstrom et al. 2009), a phenomenon well documented in many chronic pain conditions (Nees and Becker 2018; Rogers and Farris 2022; Turk and Okifuji 2002), but less so in the context of stroke.

Imaging studies suggest that lesion location—such as within the ascending somatosensory pathways (Hong et al. 2010; Elias et al. 2020), specifically spinothalamic tract and thalamus (Kim et al. 2022; Klit et al. 2011; Krause et al. 2016), basal ganglia (Klit et al. 2011; Liampas et al. 2020), brainstem (Klit et al. 2011; Krause et al. 2016; Kumar and Soni 2009), midbrain (Convers et al. 2020), insular and somatosensory cortex (Kumar and Soni 2009)—play a central role in the development of pain following stroke. This is particularly well‐described for central neuropathic PSP (Elias et al. 2020). Importantly, regions implicated in PSP partly overlap with brain systems involved in affective and interoceptive processing. In particular, the insula and anterior cingulate cortex contribute to interoception, salience processing and affective‐motivational dimensions of pain (Wager et al. 2013). These regions are also involved in anxiety and affective disorders. This overlap makes it unlikely that the underlying pathophysiology of post‐stroke pain can be disentangled or characterized solely on the basis of individual lesion locations.

Recent advances in neuroimaging have shifted the focus from isolated brain regions to whole‐brain network analyses, using methods such as lesion network mapping (LNM) (Fox 2018; Boes et al. 2015). This technique allows for a more comprehensive understanding of how disruptions in specific networks contribute to the development of a specific symptom, such as PSP. Studies using this method have highlighted the role of networks involving the thalamus, insular–opercular cortex, and cingulate cortex in central neuropathic PSP specifically, interpreting the resulting network as pain‐associated rather than pain‐specific (Elias et al. 2020; Kim et al. 2022). Moreover, the method has also shown promise in guiding targeted treatments (Elias et al. 2020). Despite advances in LNM, studies across all PSP subtypes based on larger prospective, well‐characterized ischemic stroke cohorts are still lacking.

Therefore, we set out to investigate in a comprehensive dataset of ischemic stroke patients (1) the clinical factors associated with the development of new severe pain. Furthermore, we aimed to (2) identify regions associated with the development of new and severe pain post‐stroke using classical lesion symptom mapping (LSM) and (3) identify underlying network effects associated with new and severe pain using LNM with a normative functional connectome. Ultimately, the overarching aim of the current study was to deepen our understanding of the underlying pathophysiology of PSP.

## 2 Methods

### 2.1 Study Design and Patient Population

This study utilized data from the INSPiRE‐TMS trial (NCT01586702), a multicenter, randomized trial comparing a secondary prevention support program and conventional care in patients with minor ischemic stroke or transient ischemic attack (TIA). Details of the study design and primary outcomes have been published previously (Ahmadi et al. 2020; Leistner et al. 2013). The aim of the trial was to assess whether an intensified secondary prevention program after minor stroke and TIA could reduce the frequency of recurrent vascular events. The study population included adult patients (≥ 18 years old) with a TIA (with an ABCD2 score ≥ 3) or a minor stroke (with a modified Rankin Scale (mRS) score ≤ 2) and at least one modifiable risk factor such as hypertension, diabetes, atrial fibrillation, or smoking. Exclusion criteria included malignant diseases with a life expectancy < 3 years, relevant cognitive deficits, substance dependency, or strokes/TIA caused by less common mechanisms such as dissection or vasculitis. The trial was approved by the Ethics Committee of Charité Universitätsmedizin Berlin (EA2/084/11) and by the local ethics committees at all participating centers. All patients provided written informed consent.

For this analysis, only patients with the diagnosis “minor stroke” and with information on stroke severity, as assessed by the National Institutes of Health Stroke Scale (NIHSS), were included. This study is reported in accordance with the STROBE guidelines.

### 2.2 Clinical Assessment and Follow‐Up

Patients underwent clinical assessments at baseline (within 14 days of stroke onset), 1 year (V_1_), 2 years (V_2_), and 3 years (V_3_) post‐stroke. Demographic and clinical characteristics, including age, sex, and known cerebrovascular risk factors, were documented by the primary treating physician. Stroke severity was assessed using the NIHSS. Functional outcomes were evaluated using the modified Rankin Score (mRS). Quality of life was assessed through the EuroQoL 5‐Dimension 3‐Level (EQUATION 5D‐3L) questionnaire, which includes dimensions for mobility, self‐care, daily activities, pain/discomfort, and anxiety/depression. Each dimension is scored on a scale of 1 (no problems) to 3 (extreme problems).

The main outcome of the current secondary exploratory study was ‘new severe pain’ at V_1_ (1 year follow‐up) defined by the presence of severe pain at V_1_ (EQUATION 5D‐3L = 3) but not at baseline (EQUATION 5D‐3L < 3 at baseline) (Ali et al. 2023).

### 2.3 Imaging

All patients who received an MRI scan within the acute hospital stay (within 7 days of stroke onset) underwent a standard stroke imaging protocol on a 3‐Tesla Siemens scanner. Sequences included a T2*‐weighted sequence, diffusion‐weighted imaging (DWI), “time‐of‐flight” MR angiography, and a fluid‐attenuated inversion recovery (FLAIR) sequence. The DWI protocol consisted of images acquired with a *b*‐value of 1000 s/mm (Ali et al. 2023), non‐diffusion‐weighted images (*b* = 0 s/mm (Ali et al. 2023)), and an apparent diffusion coefficient map.

### 2.4 Neuroimaging Analysis

#### 2.4.1 Preprocessing

Lesion delineation was manually performed by a trained rater (A.S.R.) on DWI‐TRACE and FLAIR sequences and supervised by at least two experienced neurologists and/or radiology residents (A.Ku., A.Kh., R.G., B.B.), all blinded to clinical data, using MRIcron (https://www.nitrc.org/projects/mricron). Further preprocessing and analyses were conducted as described previously in detail (Rangus et al. 2024). Briefly, DWI (b1000) and FLAIR lesion masks were co‐registered to skull‐stripped and bias‐corrected DWI (b0) and FLAIR sequences by FMRIB Software Library FSL (Jenkinson et al. 2012) and subsequently normalized to a standard space based on the Montreal Neurological Institute (MNI152 atlas, 1 × 1 × 1 mm) (Fonov et al. 2009) using a series of linear and nonlinear registrations (SyN algorithm) in Advanced Normalization Tools in Python (ANTsPy) (Avants et al. 2011). Following spatial normalization, a quality check was performed by overlaying the normalized lesions onto individual normalized brains to confirm proper alignment and comparing both MRI modalities (DWI and FLAIR), the normalized lesion mask with the best quality was selected for further analyses. All imaging analyses (LSM and LNM) were performed on a subgroup of patients, namely those that had available MR‐imaging with lesions co‐registered to MNI space as well as available pain data assessed via the EQUATION 5D‐3L at V_1_ (*N* = 391) (Figure S1).

#### 2.4.2 Lesion Symptom Mapping

Voxel‐wise and atlas‐based LSM analyses were performed using NiiStat (https://www.nitrc.org/projects/niistat/) to identify structural lesions associated with “new severe pain” at V_1_. A minimum lesion overlap of 5% was required and lesion volume was included as a covariate to account for its potential confounding effects (DeMarco and Turkeltaub 2018). The voxel‐wise analysis involved 10,000 permutations to ensure robust statistical testing, with family‐wise error (FWE) correction applied and statistical significance set at *p* < 0.05. An atlas‐based analysis was also performed using the Juelich Histological Atlas to enhance interpretability by linking findings to specific neuroanatomical regions, and to identify signals potentially too subtle to reach significance in the voxel‐wise analysis (Lugtmeijer et al. 2021).

#### 2.4.3 Lesion Network Mapping

The LNM analyses followed methods by Fox et al. (Fox 2018), and also as recently published by Rangus et al. (2024), using normalized binary lesion masks as seeds to assess functional connectivity within a normative connectome derived from 1000 healthy fMRI scans (Holmes et al. 2015). A connectivity profile was calculated for each patient, between voxels within the lesion mask and all other brain voxels, producing Pearson correlation coefficients averaged across the 1000 brains. Fisher z‐transformed connectivity profiles were used for further analysis. Non‐parametric permutation testing was performed with FSL *randomize* using a two‐sample *t*‐test to identify statistically significant lesion connections associated with “new severe pain” at V_1_. Pain scores were included in the analysis in a binarized manner. The analysis was run for 5000 permutations for two contrasts examining connections linked to presence [1] and absence [−1] of “new severe pain” at V_1_. FWE correction to account for multiple comparisons and threshold‐free cluster enhancement (TFCE) were applied. A threshold for significance was defined at pFWE < 0.05. Additionally, as part of a sensitivity analysis, anxiety was included as a covariate. Results were overlaid on structural atlases (Harvard‐Oxford Cortical Structural Atlas, Harvard‐Oxford Subcortical Structural Atlas, JHU White‐Matter Tractography Atlas, FLIRT‐normalized Cerebellar Atlas) to pinpoint connected regions and were compared with existing neuroimaging studies using the ‘neurosynth decoder’ on the Neurosynth platform (Gorgolewski et al. 2016).

### 2.5 Statistical Analysis

STATA IC version 17 (StataCorp, College Station, TX, USA) was used to perform all statistical analyses. Descriptive statistics were used to summarize demographic and clinical characteristics. *T*‐tests or Chi‐square (*χ* ^2^) tests were performed for group comparisons where appropriate. Bivariate and multiple logistic regression analyses were performed to investigate the associations between “new severe pain” at V_1_ and age, sex, lesion volume, NIHSS, mRS at baseline, EQUATION 5D‐3L anxiety at baseline and EQUATION 5D‐3L mobility at baseline. These variables were selected based on their clinical relevance and prior evidence linking them to pain outcomes in similar patient populations. A two‐sided statistical significance level of 0.05 was considered. However, no adjustment for multiple testing was applied in this secondary exploratory study for the regression models. Interpretation of our results is based on effect size estimates and 95% confidence intervals (CI).

## 3 Results

### 3.1 Cohort Description

A total of 1022 patients from the INSPiRE‐TMS cohort were included in the analysis. The mean age of the cohort was 66.3 years and 68.3% of the patients were male. Median NIHSS on admission was 2 (IQR 1–3), median mRS at baseline was 1 (IQR 1–2), and median ischemic brain lesion volume was 8.7 mL (IQR 2.7–33.9). Patient demographics as well as stroke‐specific parameters were similar between the entire study population and the sub‐group cohort used for subsequent LSM and LNM analyses (Table 1).

**TABLE 1:** Cohort characteristics.

	Total cohort	Subgroup with lesion registration and pain data at V_1_
Age, mean (SD)	66.3 (10.5)	66.6 (10.9)
Sex, male, *n* (%)	698 (68.3)	272 (69.6)
Hypertension, *n* (%)	840 (82.4)	307 (78.5)
Diabetes, *n* (%)	234 (22.9)	94 (24.0)
Smoker, *n* (%)	674 (66.0)	256 (65.5)
Cardiac insufficiency, *n* (%)	49 (4.9)	21 (5.5)
History of heart attack, *n* (%)	70 (7.0)	26 (6.8)
Atrial fibrillation, *n* (%)	94 (9.4)	36 (9.4)
History of stroke, *n* (%)	171 (17.0)	76 (19.8)
History of TIA, *n* (%)	59 (5.9)	24 (6.4)
TOAST classification		
Large‐artery atherosclerosis (%)	159 (15.7)	64 (16.4)
Cardioembolic stroke (%)	159 (15.7)	59 (15.1)
Small vessel occlusion (%)	191 (18.8)	62 (15.9)
Other (%)	19 (1.9)	7 (1.79)
Unknown (%)	487 (48.0)	198 (50.8)
NIHSS at admission, median (IQR)	2 (1–3)	2 (1–3)
mRS, median (IQR)	1 (1–2)	1 (1–2)
Lesion volume, in mL, median (IQR)	8.7 (2.7–33.9)	8.3 (2.7–42.4)
Total	1022	391

Abbreviations: mRS: modified Rankin Scale; NIHSS: National Institutes of Health Stroke Scale; TIA: transient ischemic attack; TOAST: Trial of Org 10,172 in Acute Stroke Treatment.

Of the 1022 patients included, 91% completed the EQUATION 5D‐3 L pain dimension questionnaire at baseline (Table S1). Pain (EQUATION 5D‐3L ≥ 2) was present in 43.1% at baseline (severe pain 3.1%) and increased by V_1_; about 5%–6% reported severe pain at follow‐up (Table S1). The subgroup with imaging data available exhibited similar pain prevalence trends (Table S1).

### 3.2 Factors Associated With New Severe Pain

Of those 39 patients with severe pain at V_1_, 31 patients experienced “new severe pain” post‐stroke, defined as patients who reported severe pain at V_1_ (EQUATION 5D‐3L = 3) but not at baseline (EQUATION 5D‐3L < 3 at baseline). In a two‐group analysis comparing patients with “new severe pain” (*N* = 31) at V_1_ to those without “new severe pain” (*N* = 745), there was no statistically significant difference in terms of basic patient demographics (age, sex) or stroke‐specific factors (cardiovascular risk factors, NIHSS, mRS, lesion volume, distribution of acute neurological deficits), quality of life measurements, and depression scales (Table S2).

Multivariable binary logistic regression identified baseline anxiety (assessed as 1, 2 or 3 in the EQUATION 5D‐3L questionnaire for anxiety) as being associated with “new severe pain” at V_1_ (odds ratio 2.9, 95% CI 1.17–7.17, *p* = 0.021). The model included age, male sex, baseline lesion volume, NIHSS and EQUATION 5D‐3L mobility as well; all these factors showed a weaker association with “new severe pain” at V_1_ than anxiety (Table 2).

**TABLE 2:** Multivariable binary logistic regression analysis for “new severe pain” at V_1_.

Multivariable binary logistic regression analysis for “new severe pain” (3) at V_1_					
	Bivariate model			Multivariable model	
	Odds ratio (95% CI)	*p*	Number of obs	Odds ratio (95% CI)	*p*
Age	0.98 (0.95–1.01)	0.43	776	0.99 (0.94–1.04)	0.63
Sex, male	0.65 (0.28–1.53)	0.32	776	0.64 (0.17–2.46)	0.52
Lesion volume	0.99 (0.92–1.07)	0.83	291	0.99 (0.91–1.07)	0.74
NIHSS	1.05 (0.93–1.20)	0.41	776	0.95 (0.70–1.30)	0.76
mRS at baseline	1.42 (0.86–2.33)	0.17	776	1.42 (0.57–3.56)	0.45
EQUATION 5D‐3L anxiety at baseline	1.90 (1.00–3.58)	**0.048**	721	2.90 (1.17–7.17)	**0.021**
EQUATION 5D‐3L mobility at baseline	1.44 (0.66–3.14)	0.35	721	2.83 (0.88–9.13)	0.08

*Note:* 776 patients included, 31 having “new severe pain” at V_1_. Number of observations in multivariable model = 270. Bold values indicate statistically significant results (*p* < 0.05).

Abbreviation: EQUATION 5D‐3L: European Quality of Life 5 Dimensions 3 Level Version.

### 3.3 Neuroimaging Results

In a subgroup analysis of 391 patients for whom MRI data was available and could be used for LSM and LNM analyses, 18 (4.6%) reported “new severe pain” at V_1_. To assess potential selection bias in the imaging subgroup, we compared patients with new severe pain who were included in the LSM/LNM analyses (*n* = 18) with those who could not be included because MRI data were unavailable (*n* = 13, Table S3). Available demographic and clinical variables were broadly comparable between groups. However, patients without lesion registration showed a higher descriptive proportion of lacunar infarcts. Lesion volume could not be compared because this variable was only available after lesion registration.

**FIGURE 1:**
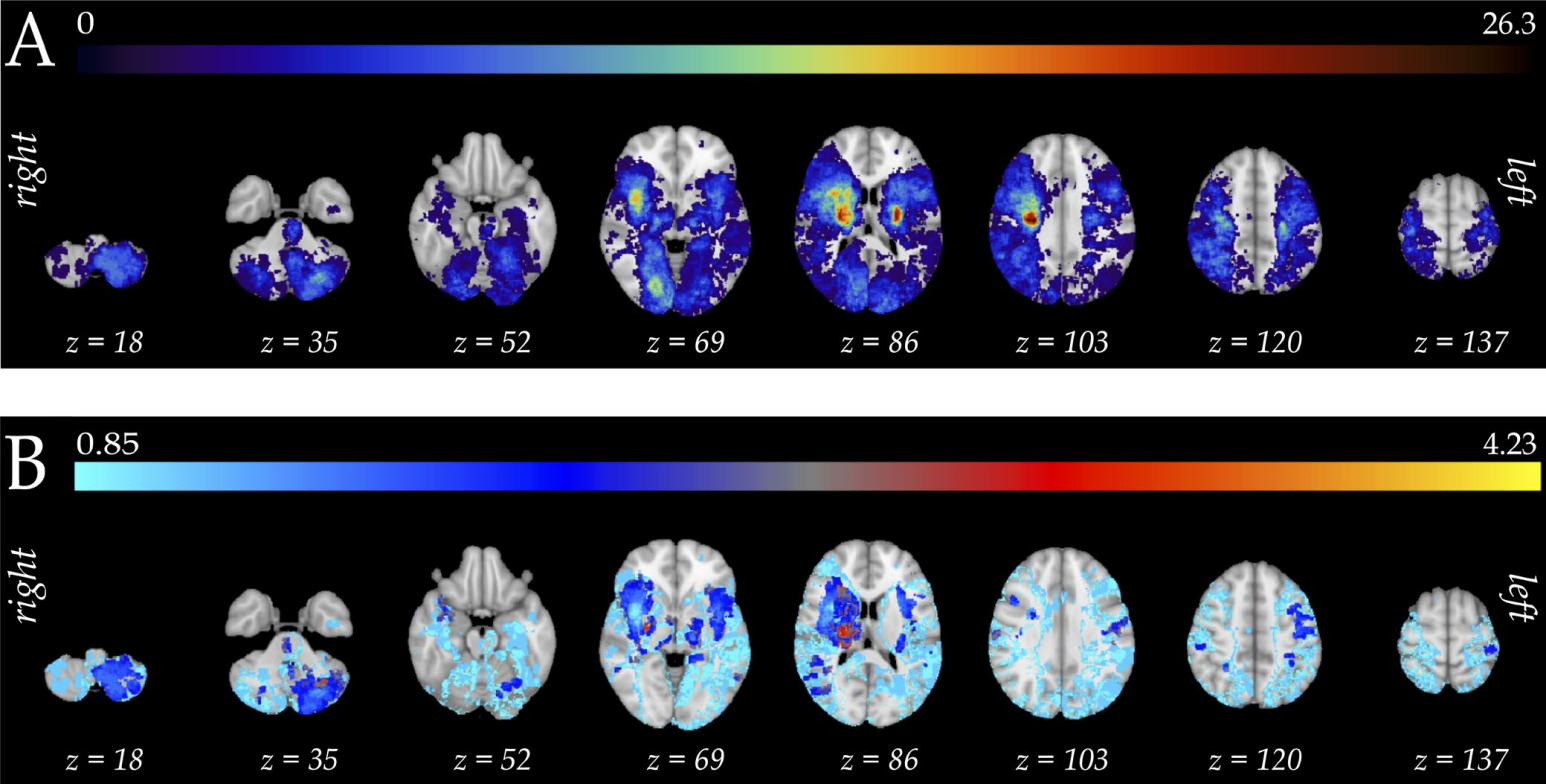
Lesion distribution and lesion symptom mapping for “new severe pain”. (A) Lesion distribution map, overlap of all lesion maps (*N* = 391). Color scale indicates the number of patients showing a lesion in a given voxel; (B) results of LSM analysis relating lesion location to “new severe pain” at V_1_ (*N* = 18 patients); The color bar represents the strength of the statistical association of a lesion location with “new severe pain”. Lesion masks and results were overlaid on slices of an ex vivo MNI space template. No voxels survived the significance threshold in LSM for “new severe pain”.

A heat map of all analyzed lesions of these patients is shown in Figure 1A. The LSM analysis for “new severe pain” at V_1_ did not reveal any voxels exceeding the significance threshold (*p* < 0.05) in neither the voxel‐wise nor atlas‐based analyses. Voxel‐wise LSM revealed weak and statistically non‐significant associations with lesions in the occipital cortex, thalamus, and cerebellum, predominantly in the right hemisphere (Figure 1B, Table S4).

#### 3.3.1 Lesion Network Mapping

**FIGURE 2:**
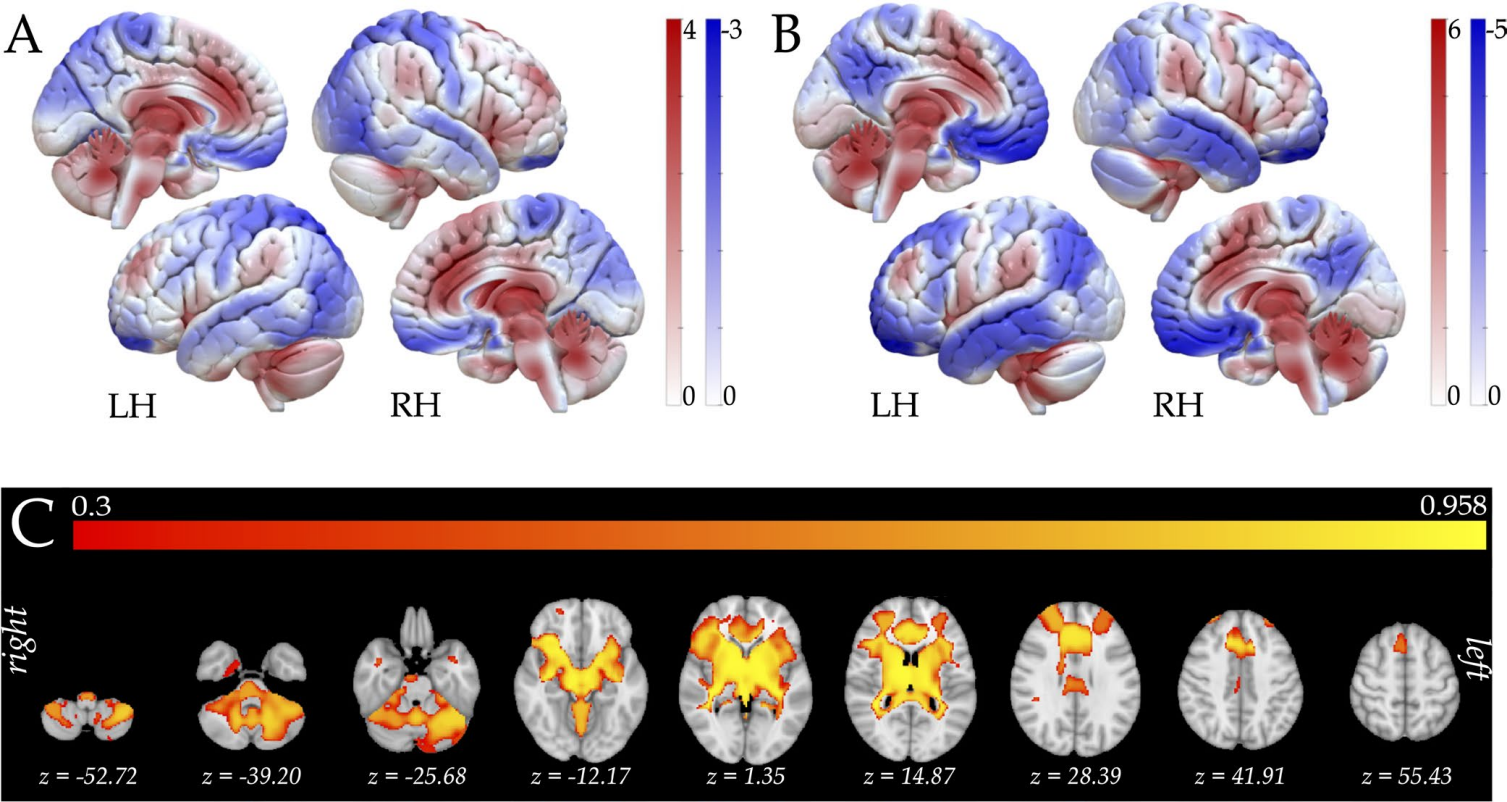
Lesion network mapping for “new severe pain” (A) Maps derived from permutation analysis using “new severe pain” (*N* = 18 patients) as the variable of interest overlaid on a 3D surface of the brain, positive correlations shown in red, negative correlations shown in blue; (B) LNM result for “new severe pain” at V_1_ with anxiety as a covariate (*N* = 18 patients); (C) Voxels associated with “new severe pain” at V_1_ (*N* = 18 patients). FWE‐corrected map thresholded at *p* < 0.05 (1‐p shown). LH indicates left hemisphere; RH, right hemisphere.

The LNM analysis of “new severe pain” at V_1_ showed disruptions to a widespread bi‐hemispheric network of regions including connections to cortical, subcortical, and cerebellar regions (Figure 2A,C). The network specifically included the frontal pole, cingulate gyrus, paracingulate gyrus, insular cortex, brainstem, thalamus, putamen, anterior thalamic radiation, and mainly crus I and VI of the cerebellum (Table 3). No regions were depicted using the significance threshold of pFWE < 0.05. A sensitivity analysis adjusting for anxiety as a covariate revealed a network similar to “new severe pain” alone (Figures 2B and S2A), with highest connectivity values to insular cortex, frontal operculum cortex, thalamus, anterior thalamic radiation, crus VI and I of the cerebellum (Figure S2B, Table 3). Regions that were depicted using the significance threshold of pFWE < 0.05 included, among others, insular cortex, thalamus, putamen, pallidum, caudate, anterior thalamic radiation, inferior fronto‐occipital fasciculus, corticospinal tract, and various cerebellar regions (Table 3). An additional sensitivity analysis of patients who have had severe pain at V_1_ (EQUATION 5D‐3L = 3) but did not report any pain at baseline (EQUATION 5D‐3L = 0) (*N* = 5 patients) was conducted. This analysis showed a similar network (Figure S2C) to the “new severe pain” network (Figure 2C), with overlapping regions particularly in cingulate cortex and insular cortex.

**TABLE 3:** Atlas based labeling of lesion network mapping results anatomical description.

	Harvard‐Oxford Cortical Structural Atlas	Harvard‐Oxford Subcortical Structural Atlas	JHU White‐Matter Tractography Atlas	Cerebellar Atlas (norm. with FLIRT)
New Severe pain Network	Frontal Pole Cingulate Gyrus, anterior division Paracingulate Gyrus Insular Cortex Frontal Orbital Cortex Frontal Operculum Cortex Superior Frontal Gyrus Inferior Frontal Gyrus, pars triangularis	Brain‐Stem Left Thalamus Right Thalamus Right Putamen Left Putamen Right Caudate Left Caudate Right Lateral Ventricle Left Lateral Ventricle Right Pallidum Left Pallidum	Anterior thalamic radiation L Anterior thalamic radiation R Forceps minor Inferior fronto‐occipital fasciculus R Inferior fronto‐occipital fasciculus L Corticospinal tract R Corticospinal tract L	Left Crus I Left VI Left Crus II Right VI Right I‐IV Right Crus I Left VIIIa Left I‐IV Right V Left VIIb Left V Right VIIIa
New Severe pain, adjusted for anxiety Network	Insular Cortex Cingulate Gyrus, anterior division Frontal Pole Paracingulate Gyrus Frontal Operculum Cortex Central Opercular Cortex Juxtapositional Lobule Cortex (formerly Supplementary Motor Cortex) Frontal Orbital Cortex	Brain‐Stem Right Thalamus Left Thalamus Right Putamen Left Putamen Right Caudate Left Caudate Right Lateral Ventricle Left Pallidum Right Pallidum Left Lateral Ventricle	Anterior thalamic radiation L Anterior thalamic radiation R Inferior fronto‐occipital fasciculus R Corticospinal tract R Corticospinal tract L Inferior fronto‐occipital fasciculus L	Left VI Right VI Left Crus I Left VIIIa Right V Left V Right I‐IV Right VIIIa Left VIIb Left I‐IV Left VIIIb Right VIIIb Vermis VI Right Crus I Left Crus II Right VIIb
New Severe pain, adjusted for anxiety Network (Voxels *p* > 0.95)	Insular Cortex	Right Thalamus Left Thalamus Right Putamen Left Putamen Left Pallidum Right Pallidum Right Caudate Right Lateral Ventricle Brain‐Stem Left Caudate	Anterior thalamic radiation L Anterior thalamic radiation R Inferior fronto‐occipital fasciculus R Inferior fronto‐occipital fasciculus L Corticospinal tract R Corticospinal tract L	Left VI Left Crus I Left VIIIa Left VIIb Left V Right V Vermis VI

### 3.4 Comparison to Neurosynth

The decoding tool available on the Neurosynth platform which encompasses data from 14,371 studies (https://www.neurosynth.org) was used to systematically compare the spatial similarity of our identified networks with all the maps in its database.

**FIGURE 3:**
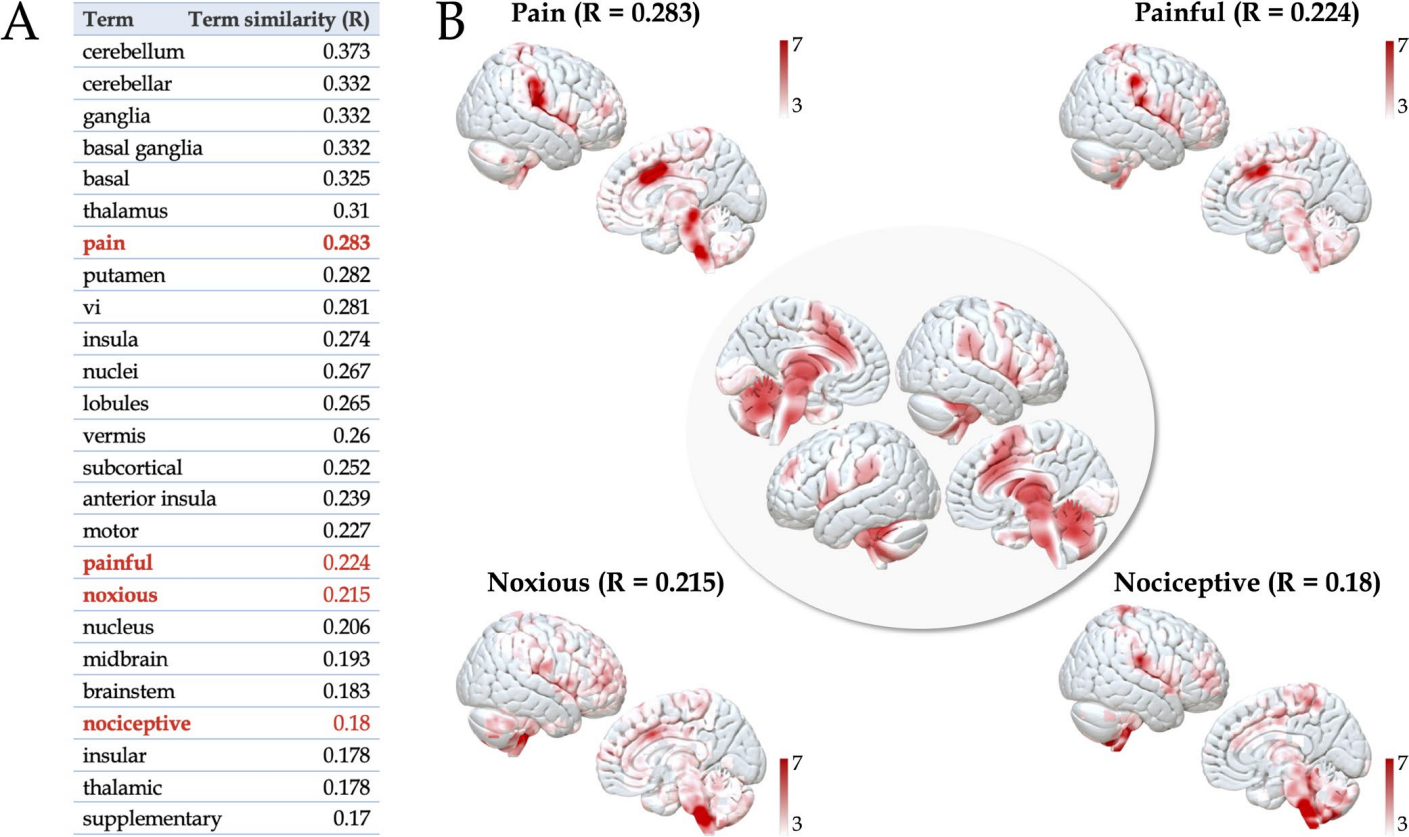
“New severe pain” network compared to association maps from Neurosynth. (A) Top 25 associated terms to “new severe pain” (with anxiety as a covariate), purely anatomical terms are shown in black, non‐anatomical terms shown are in red; (B) in the middle depiction of LNM results of “new severe pain” with anxiety as covariate, around it association test maps of non‐anatomical terms from Neurosynth decoder results overlaid on a brain surface. Color bar indicates *T*‐score.

Out of 1334 anatomical and functional terms, Neurosynth comparisons revealed that the top 25 similarities to the unthresholded “new severe pain with anxiety as covariate” map (Figure 2B) included terms specifically associated with “*pain*”, “*painful*”, “*noxious*” and “*nociceptive*” (Figure 3A,B). In contrast, the “new severe pain” showed top 25 similarities exclusively related to anatomical terms (Table S5).

## 4 Discussion

This study provides new insights into the prevalence and characteristics of post stroke pain (PSP) and the neural correlates of PSP, highlighting the complex interplay between psychological factors, lesion characteristics, and brain network dysfunction. These findings contribute to a growing understanding of PSP as a complex condition requiring a multidisciplinary approach to treatment and rehabilitation.

In our cohort, up to 50% of stroke survivors reported any PSP, with severe pain affecting 5%–6% of patients. These findings align with previous studies (Ali et al. 2023; Hansen et al. 2012; Naess et al. 2012a), which observed similar pain prevalence despite differences in stroke severity. Notably, the mild median NIHSS score in our cohort emphasizes that PSP is not solely a consequence of severe strokes. Our results further suggest an association between psychological factors and pain outcomes, identifying anxiety as the main characteristic associated with new severe PSP. This is consistent with the well‐documented role of psychological factors in different chronic pain conditions (Nees and Becker 2018; Rogers and Farris 2022; Turk and Okifuji 2002), but has not been described in PSP so far. Notably, Naess et al. (2012b) reported pain, depression, and fatigue as a symptom‐cluster arising after stroke. These findings underscore the need for early psychological assessment and intervention. Unaddressed psychological comorbidities may exacerbate PSP, impede recovery, and contribute to adverse outcomes, as chronic pain itself is associated with higher 10‐year mortality (Torrance et al. 2010).

Neuroimaging analyses were performed on a subgroup closely resembling the total cohort in terms of patient‐specific characteristics, including a mild median NIHSS. Although voxel‐ and atlas‐based LSM analyses did not yield statistically significant results, they identified weak and statistically not significant associations between severe pain and lesions in the occipital cortex, thalamus, and cerebellum. This aligns with prior evidence linking these regions to PSP (Elias et al. 2020; Kim et al. 2022).

However, the notion of a single “pain region” in the brain is overly simplistic. Pain perception is a complex cognitive process involving multiple regions and networks, often referred to as the “neurological signature of physical pain” (Wager et al. 2013), with significant inter‐subject variability influenced by previous pain experiences and psychological states (Nees and Becker 2018).

Despite the generally mild stroke severity of patients included in our study with corresponding small ischemic brain lesion sizes, LNM revealed a coherent network associated with PSP, identifying disruptions in a distributed network encompassing the anterior cingulate cortex (ACC), insular cortex and thalamus. Adjusting our LNM analyses for anxiety refined the pain network further, underscoring the interactive effects of psychological states on pain perception. Importantly, pain and anxiety are clinically and neurobiologically intertwined, and cingulo‐insular and thalamic regions may contribute to sensory‐discriminative, affective‐motivational, and interoceptive components of pain. At the same time, prior lesion network mapping studies of affective and neuropsychiatric symptoms have identified networks that are not identical to the pain‐related network observed here (Rios et al. 2025; Siddiqi et al. 2024, 2020), supporting the possibility that partially distinct lesion‐related circuits contribute to pain and affective symptoms. Notably, the insular cortex and ACC play a central role in the affective and valence‐related dimensions of chronic pain. The insular cortex is implicated in interoceptive awareness (Labrakakis 2023; Lu et al. 2016), while the ACC is associated with pain's aversive and cognitive components (Fuchs et al. 2014; Xiao and Zhang 2018) and is an established target in neuromodulatory pain therapies (Xiao et al. 2021; Motzkin et al. 2023). Being involved in the limbic system, it may be particularly effective as a therapeutic target in pain patients with affective comorbidities, though further research is warranted. The thalamus, a structurally and functionally multifaceted region, acts as a key hub in our severe pain network. Its ventral posterolateral (VPL) and ventral posteromedial (VPM) nuclei transmit nociceptive information to the somatosensory cortex, influencing both the sensory‐discriminative aspects of pain perception (Cao et al. 2024; Kuner and Kuner 2021), and pain intensity itself (You et al. 2022). Additionally, the intralaminar nuclei and mediodorsal nucleus, connected to limbic structures such as the ACC (Kuner and Kuner 2021) and the primary motor cortex (Gan et al. 2022), further modulate the affective‐motivational and motor components of pain (Kuner and Kuner 2021). The involvement in pain processing is supported by clinical studies demonstrating that thalamic neuromodulation, specifically targeting the VPM/VPL nuclei, has been used therapeutically to alleviate chronic pain (Motzkin et al. 2023; Tan et al. 2023).

Overall, these findings support the notion that PSP is linked not to distinct lesion locations but rather to disruptions within a functionally connected defined network, reinforcing the view that neuromodulatory pain therapy should target networks rather than single regions (Motzkin et al. 2023).

Although we could not differentiate for pain etiology (vide infra)—given the limited information on pain character and onset—our results overlap with those reported by Elias et al. (2020), who investigated solely central neuropathic PSP. Their findings similarly implicated the insular cortex, thalamus, and ACC in PSP, supporting the plausibility of our results and emphasizing the strong interaction with specific disrupted networks and pain perception independent of pain etiology. Neurosynth comparisons support this notion, as our *pain + anxiety* network most strongly overlapped with pain‐related terms that were defined meta‐analytically and without etiologic context. Neurosynth decoding should be interpreted cautiously because it relies on meta‐analytic term associations and reverse inference. However, it provides a transparent, data‐driven way to contextualize whether the observed network resembles previously reported pain‐related activation patterns.

Approximately two‐thirds of patients with central PSP do not receive adequate pain therapy (Haslam et al. 2021), despite evidence that effective pain treatment is associated with improvements in cognition and quality of life (Harrison and Field 2015). Although severe pain was present only in a smaller subgroup of patients, its occurrence even after predominantly minor ischemic stroke highlights the importance of systematic pain assessment during follow‐up. The association between PSP and anxiety further supports the need to investigate a multidisciplinary approach to pain management. Traditional treatments focusing solely on somatic pain mechanisms may be insufficient, particularly in patients with psychological comorbidities. Adequate pain therapy after stroke should therefore not be limited to analgesic medication alone, but may require multidisciplinary management including psychological support, rehabilitation, and treatment of affective comorbidities if present. Moreover, longitudinal studies are needed to evaluate how optimized pain therapy may affect long‐term outcomes and stroke recurrence. Understanding the bidirectional relationship between pain and psychological states could further refine treatment protocols and improve quality of life for stroke survivors.

Neuromodulation may offer additional relief, considering our findings of network disruptions associated with severe pain. LNM findings could help define both invasive and non‐invasive neuromodulation targets for PSP patients with or without anxiety—a strategy that has proven successful for other symptoms (Weigand et al. 2018; Joutsa et al. 2018). Further studies are now needed to validate these network targets.

Key strengths of the present study include its large, well‐characterized cohort stemming from a randomized controlled trial and the integration of both clinical and neuroimaging data. The use of LNM provided a network‐level perspective on PSP, offering novel insights into its neural underpinnings. By including patients with heterogenous lesion locations—both cortical and subcortical—we minimized bias related to lesion site, a strength compared to other pain‐LNM studies that, for example, focused specifically on thalamic lesions (Kim et al. 2022).

### 4.1 Limitations

An important limitation is the study's secondary, exploratory nature, which provided only limited information on pain quality, etiology, and potential pre‐existing pain syndromes. We were hence unable to make inferences about pain etiology in our cohort. Future prospective studies should address these important gaps by incorporating comprehensive assessments of pain characteristics, pre‐stroke pain history, pain medication usage, as well as the impact of comorbid conditions, such as depression and anxiety disorders, on PSP. Because the cohort did not include a non‐stroke hospitalized control group, we cannot fully disentangle lesion‐related network effects from broader consequences of acute illness, hospitalization, or early post‐stroke psychological distress. In addition, 13 patients with new severe pain could not be included in the imaging analysis because MRI data were unavailable. A residual selection bias cannot be excluded, particularly with respect to lesion volume. Therefore, the LNM findings may not fully generalize to all patients with new severe pain in the cohort. A further important limitation is the small number of patients with new severe pain. Although the overall cohort is large, the number of patients meeting the strict definition of new severe pain is small. This limited event number reduces statistical power and increases the risk of both false‐negative findings, particularly in LSM, and false‐positive or unstable findings in voxel‐wise network analyses. Therefore, the neuroimaging findings should be interpreted as exploratory network‐level correlates that require external validation in independent cohorts. Furthermore, the use of LNM with normative connectomes provides an indirect measure of functional disruption, hence explaining symptoms with reduced variance compared to direct measures of functional connectivity. Additionally, for this type of LNM analysis, averaging of BOLD signals within the entire lesion mask is required, which can be problematic specifically in large lesions spanning gray and white matter. Prospective studies are needed to identify the specific contributions of gray and white matter in the development of pain after stroke (Salvalaggio et al. 2021). Nonetheless, this type of methodology is advantageous by facilitating the analysis of large amounts of lesion data from patients coming directly from a clinical setting in which functional MRI scanning is not part of the patient care routine, allowing us to identify what the lesioned area would be connected to in the healthy brain, potentially shedding light into causal disruptions associated with the presence of a specific symptom.

## 5 Conclusion

PSP is a significant burden for stroke survivors. It likely arises from a complex interplay of lesion location, network dysfunction, and psychosocial factors. This study has the following major findings: (1) up to 50% of stroke survivors experience any PSP, (2) anxiety emerges as a critical associated factor for new‐onset severe pain, and (3) neuroimaging reveals disruptions in a key brain network associated with PSP, including the ACC and insular cortex, providing a strong potential neuroanatomical substrate of PSP. These findings underscore the need for integrated, multidisciplinary care to address the multifaceted nature of PSP. By leveraging insights from network‐level analyses, clinicians can move toward more personalized and effective treatment strategies, ultimately improving long‐term outcomes and quality of life.

## Author Contributions

A.S. and A.Ku. designed the study and prepared the manuscript. A.S.R. delineated lesions, and A.Ku., A.Kh., R.G. and B.B. supervised lesion delineation. A.S., A.S.R. and U.T. performed the pre‐processing of the imaging data. A.S. performed regression analysis on demographic and clinical data, and ran LSM and LNM analyses. A.S.R., A.Kh., U.G., U.T., A.H., H.A. and M.E. participated in study design and interpretation of results. A.Ku. and M.E. jointly supervised this work.

## Funding

The author(s) disclosed receipt of the following financial support for the research, authorship, and/or publication of this article: A.S. and A.Ku. are participants in the Berlin Institute of Health‐Charité (Junior) Clinical Scientist Program funded by the Charité–Universitätsmedizin Berlin and the Berlin Institute of Health. M.E. received funding from the Deutsche Forschungsgemeinschaft (DFG, German Research Foundation) under Germany's Excellence Strategy‐EXC‐2049‐390688087. This project was funded by the B07 Project of the Collaborative Research Center ReTune TRR 295–424778381 (A.Ku. and M.E.). M.E. received additional funding from the Bundesministerium für Bildung und Forschung (BMBF; German Ministry for Education and Research) for the Center for Stroke Research Berlin. A.H. was supported by the National Institutes of Health (R01MH130666, 1R01NS127892‐01, 2R01 MH113929 and UM1NS132358).

## Ethics Statement

The trial was approved by the Ethics Committee of Charité Universitätsmedizin Berlin (EA2/084/11) and by the local ethics committees at all participating centers. All patients provided written informed consent.

## Conflicts of Interest

A.H. reports lecture fees for Boston Scientific and is a consultant for FxNeuromodulation and Abbott and serves as a co‐inventor on a patent application by Charité University Medicine Berlin that covers multisymptom DBS fiberfiltering and an automated DBS parameter suggestion algorithm unrelated to this work. The application has been submitted on July 21, 2023, with the patent office of Luxembourg (application #LU103178). M.E. reports grants from Bayer and Ipsen, and fees paid to the Charité from Amgen, AstraZeneca, Bayer Healthcare, BMS, Daiichi Sankyo, all outside the submitted work.

## Supporting information


**Figure S1:** Inclusion of patients from INSPiRE‐TMS trial for analysis of trajectory of pain and for neuroimaging analysis.
**Figure S2:** Lesion network mapping.
**Table S1:** Pain prevalence at different timepoints.
**Table S2:** Patient characteristics of patients with or without “new severe pain” at V_1_% indicates percentage of patient with that characteristic out of patients with new severe pain, or of all patients without new severe pain.
**Table S3:** Patient characteristics of patients with “new severe pain”, with or without lesion registration at V_1_% indicates percentage of patient with that characteristic out of patients with new severe pain, or of all patients without new severe pain.
**Table S4:** Atlas based labeling of lesion symptom mapping results anatomical description.
**Table S5:** Neurosynth results for the “new severe pain” network.

## Data Availability

Data supporting the results of this study are available upon request from the corresponding author. Open source software tools were used for the pre‐processing and analysis of the data, including: Lead‐DBS (https://github.com/netstim/leaddbs), FSL 6.0.6.4 (https://fsl.fmrib.ox.ac.uk/fsl/fslwiki/) and ANTsPy (https://github.com/ANTsX/ANTsPy).
