# Supplementary material for: Neuroimaging Correlates of Post‐Stroke Pain After Ischemic Stroke: Secondary Analysis of the INSPiRE‐TMS Trial
Source: Hum Brain Mapp. 2026 Jul 8;47(10):e70602. doi: 10.1002/hbm.70602 (PMC13345988; doi:10.1002/hbm.70602)
Supplement: Supplementary file 1 — Figure S1: Inclusion of patients from INSPiRE‐TMS trial for analysis of trajectory of pain and for neuroimaging analysis. Figure S2: Lesion network mapping. Table S1: Pain prevalence at different timepoints. Table S2: Patient characteristics of patients with or without “new severe pain” at V1% indicates percentage of patient with that characteristic out of patients with new severe pain, or of all patients without new severe pain. Table S3: Patient characteristics of patients with “new severe pain”, with or without lesion registration at V1% indicates percentage of patient with that characteristic out of patients with new severe pain, or of all patients without new severe pain. Table S4: Atlas based labeling of lesion symptom mapping results anatomical description. Table S5: Neurosynth results for the “new severe pain” network. [file HBM-47-e70602-s001.docx]

***Supplementary File***

**Neuroimaging Correlates of Post-Stroke Pain After Ischemic Stroke: Secondary Analysis of the INSPiRE-TMS Trial**

Alina Stegemann, MD^1,2,3^, Ana Sofia Rios, MD, MSc^1,2^, Ahmed Khalil, MD, PhD^1,2^, Ulrike Grittner, PhD^3,4^, Uchralt Temuulen, MSc^1,2^, Ramanan Ganeshan, MD^1,2^, Tim Bastian Braemswig, MD^1,2,3,5^, Andreas Horn, MD, PhD^6,7^, Thomas Ihl, MD^1,2^, Heinrich J Audebert, MD^1,2^, Anna Kufner, MD, PhD* ^1,2,3,8^, Matthias Endres, MD* ^1,2,5,8,9^

***these authors contributed equally.**

**Supplementary Figures:**

Suppl. Figure 1 - Inclusion of patients from INSPiRE-TMS trial for analysis of trajectory of pain and for neuroimaging analysis

Suppl. Figure 2 - Lesion network mapping

**Supplementary Tables:**

Suppl. Table 1 - Pain prevalence at different timepoints

Suppl. Table 2 - Patient characteristics of patients with or without “new severe pain” at V_1_

Suppl. Table 3 – Patient characteristics of patients with “new severe pain”, with or without lesion registration at V1

Suppl. Table 4 - Atlas based labelling of lesion symptom mapping results anatomical description

Suppl. Table 5 - Neurosynth results for the “new severe pain” network


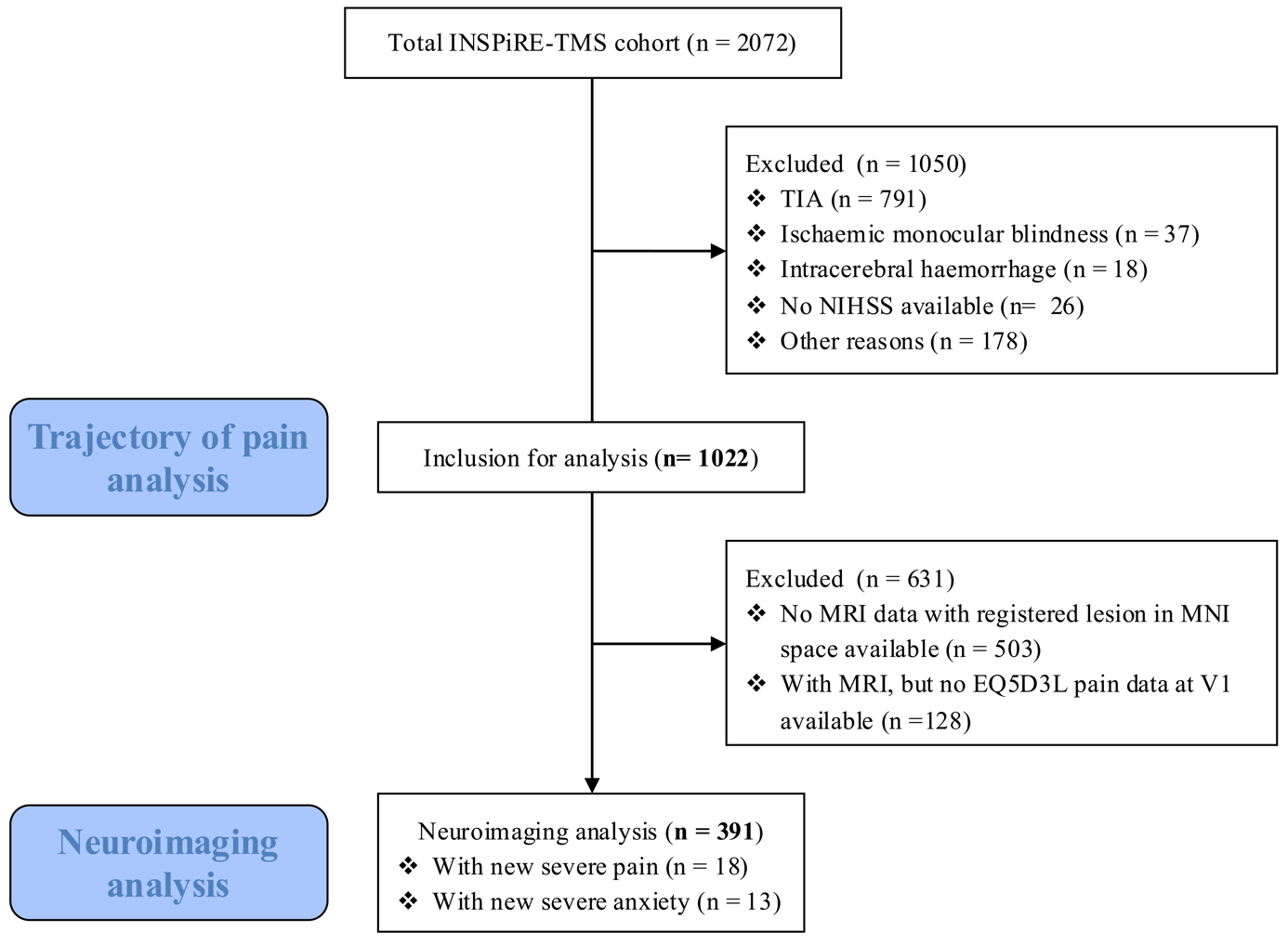


Suppl. Figure 1 – Inclusion of patients from INSPiRE-TMS trial for analysis of trajectory of pain and for neuroimaging analysis

| Total cohort (N = 1022) | | | | | | | | | |
| --- | --- | --- | --- | --- | --- | --- | --- | --- | --- |
|  | **No Pain (1)** | | **Some Pain (2)** | | **Severe Pain (3)** | | **Any Pain (≥2)** | | **Total** |
|  | N | % | N | % | N | % | N | % |  |
| Baseline | 528 | 56.9 | 371 | 40.0 | 29 | 3.1 | 400 | 43.1 | 928 |
| V_1_ | 410 | 52.8 | 327 | 42.1 | 39 | 5.0 | 366 | 47.2 | 776 |
| V_2_ | 362 | 51.1 | 302 | 42.7 | 44 | 6.2 | 346 | 48.9 | 708 |
| V_3_ | 311 | 50.5 | 272 | 44.2 | 33 | 5.4 | 305 | 49.5 | 616 |
| Patients with lesion registration and pain data at V_1_ (N = 391) | | | | | | | | | |
|  | **No Pain (1)** | | **Some Pain (2)** | | **Severe Pain (3)** | | **Any Pain (≥2)** | | **Total** |
|  | N | % | N | % | N | % | N | % |  |
| Baseline | 206 | 56.3 | 148 | 40.4 | 12 | 3.3 | 160 | 43.7 | 366 |
| V_1_ | 207 | 52.9 | 160 | 40.9 | 24 | 6.1 | 184 | 47.1 | 391 |
| V_2_ | 159 | 53.5 | 117 | 39.4 | 21 | 7.1 | 138 | 46.5 | 297 |
| V_3_ | 127 | 48.5 | 117 | 44.7 | 18 | 6.9 | 135 | 51.5 | 262 |

Suppl. Table 1 - Pain prevalence at different timepoints

|  | No or Mild Pain  (1 and 2) | New Severe Pain (3) | p-value | *Missings* |
| --- | --- | --- | --- | --- |
| *Number of patients* | *745* | *31* |  |  |
| Age, mean (SD) | 66.0 (10.6) | 64.5 (11.6) | 0.43 | */* |
| Sex, male, n (%) | 514 (69.0) | 24 (77.4) | 0.32 | */* |
| Hypertension, n (%) | 606 (81.3) | 23 (74.2) | 0.31 | *1* |
| Diabetes, n (%) | 170 (22.8) | 11 (37.9) | 0.10 | */* |
| History of smoking, n (%) | 494 (66.3) | 19 (61.3) | 0.56 | */* |
| Cardiac insufficiency, n (%) | 35 (4.8) | 0 (0) | 0.22 | *13* |
| History of Heart attack, n (%) | 45 (6.1) | 2 (7.4) | 0.95 | *15* |
| Atrial fibrillation, n (%) | 67 (9.1) | 1 (3.4) | 0.26 | *13* |
| History of Stroke, n (%) | 136 (18.5) | 3 (10.3) | 0.21 | *10* |
| History of TIA, n (%) | 50 (6.9) | 0 (0) | 0.13 | *18* |
| NIHSS at admission, median (IQR) | 2 (1-3) | 2 (1-3) | 0.41 | */* |
| mRS, median (IQR) | 1 (0-1) | 1 (0-1) | 0.84 | *141* |
| Lesion volume, in mL, median (IQR) | 8.4 (2.7-43.0) | 8.4 (1.0-45.0) | 0.83 | *633* |
| Lacunar infarct, n (%) | 212 (28.4) | 9 (29.0) | 0.99 | *17* |
| Initial paresis, n (%) | 403 (54.1) | 15 (48.4) | 0.61 | *10* |
| Initial language impairment, n (%) | 115 (15.4) | 7 (22.6) | 0.23 | *19* |
| Initial speech impairment, n (%) | 240 (32.2) | 13 (41.9) | 0.17 | *11* |
| Initial sensory impairment, n (%) | 296 (39.7) | 11 (35.5) | 0.59 | *10* |
| Initial depressed mood in past month, n (%) | 121 (16.2) | 5 (16.1) | 0.98 | *2* |
| Initial less enjoyment of favorite activities, n (%) | 80 (10.7) | 5 (16.1) | 0.35 | *1* |
| Becks Depression Scale, median (IQR) | 12 (7-18) | 20 (11.5-28.5) | 0.052 | *685* |
| EQ5D-3L Score, median (IQR) | 6 (5-7) | 5 (5-7) | 0.51 | *158* |

Suppl. Table 2 – Patient characteristics of patients with or without “new severe pain” at V_1_

% indicates percentage of patient with that characteristic out of patients with new severe pain, or of all patients without new severe pain.

|  | No lesion registration | Lesion registration | *Missings* |
| --- | --- | --- | --- |
| *Number of patients* | *13* | *18* |  |
| Age, mean (SD) | 64.4 (11.12) | 64.5 (12.3) | */* |
| Sex, male, n (%) | 10 (76.9) | 14 (77.8) | */* |
| NIHSS at admission, median (IQR) | 1 (1-3) | 2.5 (1-5) | */* |
| mRS, median (IQR) | 0 (0-1) | 1 (0-1) | *3* |
| Lesion volume, in mL, median (IQR) | / | 8.4 (1.0-45.0) | *13* |
| Lacunar infarct, n (%) | 6 (46.2) | 3 (16.6) | */* |
| Initial paresis, n (%) | 7 (53.8) | 8 (44.4) | *1* |
| Initial language impairment, n (%) | 2 (15.4) | 5 (27.8) | *2* |
| Initial speech impairment, n (%) | 4 (30.8) | 9 (50.0) | *2* |
| Initial sensory impairment, n (%) | 4 (30.8) | 7 (38.9) | */* |

Suppl. Table 3 – Patient characteristics of patients with “new severe pain”, with or without lesion registration at V1

% indicates percentage of patient with that characteristic out of patients with new severe pain, or of all patients without new severe pain.

|  | Harvard-Oxford Cortical Structural Atlas | Harvard-Oxford Subcortical Structural Atlas | JHU White-Matter Tractography Atlas | Cerebellar Atlas (norm. with FLIRT) |
| --- | --- | --- | --- | --- |
| New Severe Pain | Lateral Occipital Cortex, superior division  Precentral Gyrus  Postcentral Gyrus  Insular Cortex  Middle Frontal Gyrus  Occipital Pole  Lateral Occipital Cortex, inferior division  Lingual Gyrus  Central Opercular Cortex  Angular Gyrus  Superior Parietal Lobule  Supramarginal Gyrus, posterior division  Precuneous Cortex  Frontal Pole  Middle Temporal Gyrus, temporooccipital part  Supramarginal Gyrus, anterior division  Occipital Fusiform Gyrus  Inferior Frontal Gyrus, pars opercularis  Frontal Orbital Cortex  Frontal Operculum Cortex  Parietal Operculum Cortex  Inferior Frontal Gyrus, pars triangularis  Superior Temporal Gyrus, posterior division  Planum Temporale  Temporal Occipital Fusiform Cortex | Brain-Stem  Right Thalamus  Right Putamen  Left Thalamus  Right Caudate | Superior longitudinal fasciculus L  Anterior thalamic radiation R  Inferior fronto-occipital fasciculus R  Superior longitudinal fasciculus R | Left Crus I  Left Crus II  Left VI  Right Crus I  Right Crus II  Left VIIb  Left VIIIa  Left VIIIb  Right VI  Left IX |

Suppl. Table 4 – Atlas based labelling of lesion symptom mapping results anatomical description


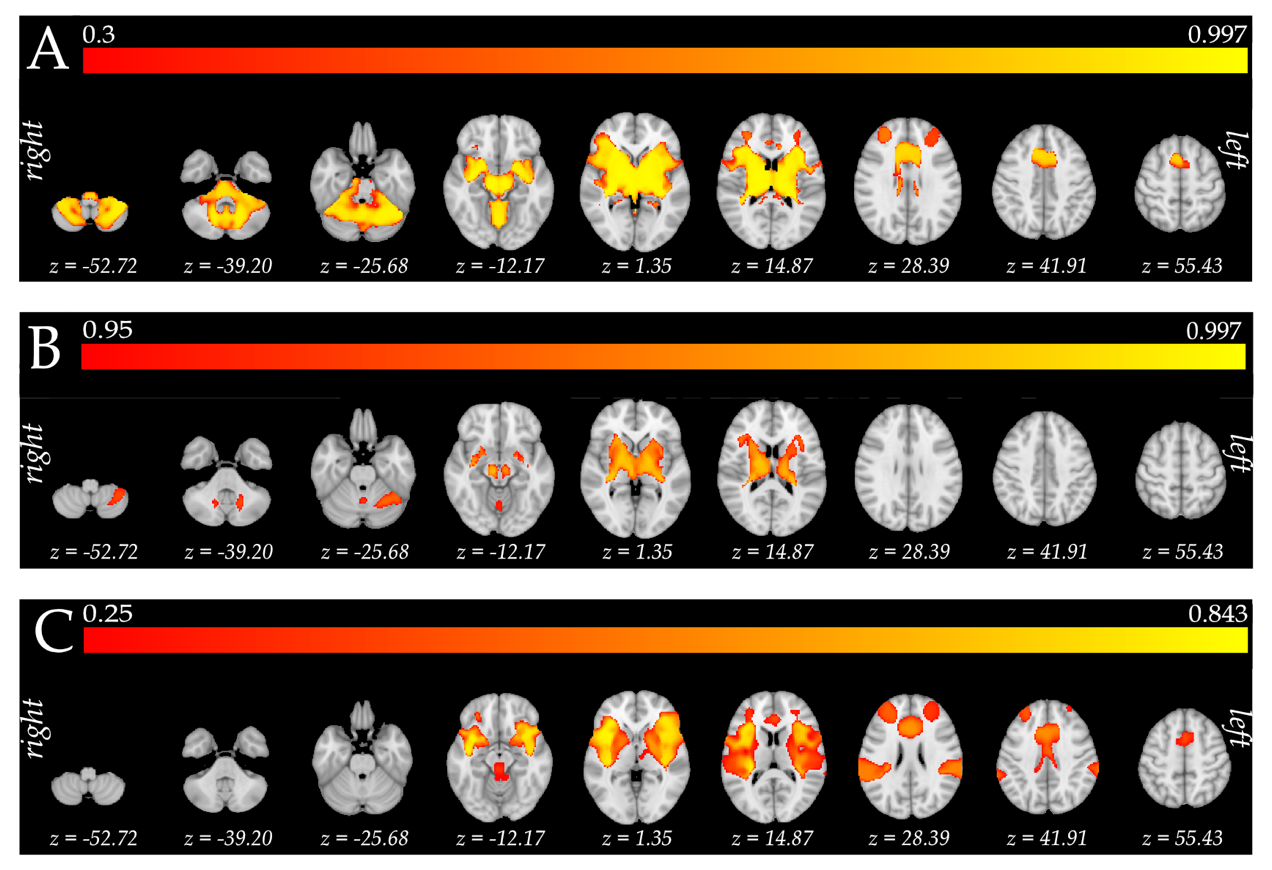


Suppl. Figure 2 – Lesion network mapping

**A:** Voxels associated with “new severe pain” with anxiety as a covariate (N = 18 patients); **B**: Voxels statistically significantly associated with “new severe pain” at V1 with anxiety as a covariate (N = 18 patients); **C**: Subgroup analysis of “new severe pain”: Voxels associated with severe pain at V_1_ (EQ5D-3L = 3) but no pain at baseline (EQ5D-3L = 0) (N = 5 patients). FWE-corrected maps thresholded at p<0.05 (1-p shown).

| Term | Term similarity (R) | |
| --- | --- | --- |
| thalamus  basal  basal ganglia  ganglia  cerebellum  cerebellar  nucleus  putamen  insula  vi  nuclei  anterior insula  subcortical  anterior  anterior cingulate  cingulate  vermis  striatal  lobules  caudate  pain  midbrain  acc  caudate nucleus  striatum | | 0.312  0.307  0.307  0.306  0.306  0.299  0.273  0.265  0.262  0.261  0.256  0.247  0.241  0.239  0.238  0.233  0.229  0.227  0.226  0.218  0.203  0.202  0.198  0.195  0.192 |

Suppl. Table 5– Neurosynth results for the “new severe pain” network
